# Locked book pubic symphysis: A case report in a resource-limited setting in sub-Sahara Africa

**DOI:** 10.1016/j.ijscr.2019.09.019

**Published:** 2019-09-25

**Authors:** O.K. Fola, M.L. Guifo, J.G. Tsiagadigui, P. Tolefac, M. Biyouma, I. Djoko, A. Essomba

**Affiliations:** a Surgical Unit, Yaoundé University Teaching Hospital, Yaoundé, Cameroon; b Department of Surgery and Subspecialties, Faculty of Medicine and Biomedical Sciences, University of Yaoundé I, PO BOX 1364, CHU, 237 Yaoundé, Cameroon; c Surgical Unit, Edéa Annex Regional Hospital, Edéa, Cameroon

**Keywords:** Locked pubic symphysis, Reduction, Case report

## Abstract

**Introduction:**

Locked pubic symphysis is a rare but serious injury that occurs by lateral compression mechanism. It is an overlapping pubic symphysis dislocation, with one pubic bone entrapped behind the contralateral pubis, locked into the contralateral obturator foramen.

**Case report:**

We report the case of a 25 years old male who presented with acute urine retention following a left lateral compression pelvic trauma by a motor vehicle and was diagnosed with a locked pubic symphysis treated successfully by open reduction and combined internal and external fixation.

**Conclusion:**

Locked pelvic symphysis is a rare form of pelvic injury. The majority of cases require stabilization by open reduction and internal fixation of the anterior pelvic ring. Urogenital injuries are the commonest associated injuries, despite unfavourable mechanism.

## Highlights

• Locked pubic symphysis is a serious injury of the pelvis.
• Very few cases has been reported in the literature.
• This nosological entity shows peculiarities in its manually difficult reduction and associated complications.
• A good outcome can be obtained after management with simple gestures, possible even in ressource-limited settings.

## 1 Introduction

A locked pubic symphysis occurs when one pubic bone becomes entrapped behind the contralateral pubis after a lateral compression mechanism of injury resulting to an overlapped pubic symphysis [1,2]. Only a very small proportion of pelvic trauma resulting from lateral compression end up as locked injuries [1]. This type of injury was first described in English literature in 1952 by Eggers [3]. To the best of our knowledge, there are less than twenty cases in English literature since it original description [2,[4], [5], [6]]. Also, there is no mention of this injury in Cameroon. We describe a locked pubic symphysis with greater than 4 cm overlap that was reduced with simple maneuvers and instruments that are readily available; in the emergency department of our institute.

These injuries often result from high energy blunt trauma, most commonly owing to motor vehicle accidents. Like all other pelvic injuries, this type of injury is usually associated with significant morbidity and mortality both from the complications of pelvic ring fractures and from other associated injuries [7]. The work has been reported in line with the SCARE criteria [8].

## 2 Presentation of case

A 25 years old male, student, with no significant medical past medical and surgical history, presented at the emergency department of our institute on the 12th October 2018, brought by non-medicalised car, referred from a dispensary by a physician, with inability to bear weight on the left side and acute urinary retention following a road traffic accident. The patient was a pedestrian involved in a road traffic crash and subsided a side on compression between a mobile truck and a fixed truck resulting to a pelvic injury. Following that, there was inability to bear weight on his left side and acute urine retention. He then consulted in a nearby dispensary where the transurethral catheterization fails prompting referral to our institute within 24 h.

There, on primary survey, the patient had patent airways with a respiratory rate of 18 cpm, then a blood pressure of 121/72 mmHg and a pulse of 76 bpm. His Glasgow coma score was 15/15 and pupils were equal and reactive to light. After secondary survey, a complete tortious examination show an enlarged bladder. There was no blood at the urethral meatus. On digital rectal examination, there was a high riding prostate. Following lock and open pelvic maneuver were painful. Our working diagnosis was closed pelvic trauma associated with acute urine retention. According to the mechanism of trauma (lateral compression) and clinical findings, we thought of transverse fracture of the pubic rami, or posterior compression of sacroiliac (SI) joint without ligament disruption (stable), or posterior SI ligament rupture, or sacral crush injury, or iliac wing fracture (rotationally unstable, vertically stable), or probably associated open book (APC) injury to contralateral pelvis (completely unstable), or overlapped pubic symphysis.

**Fig. 1:**
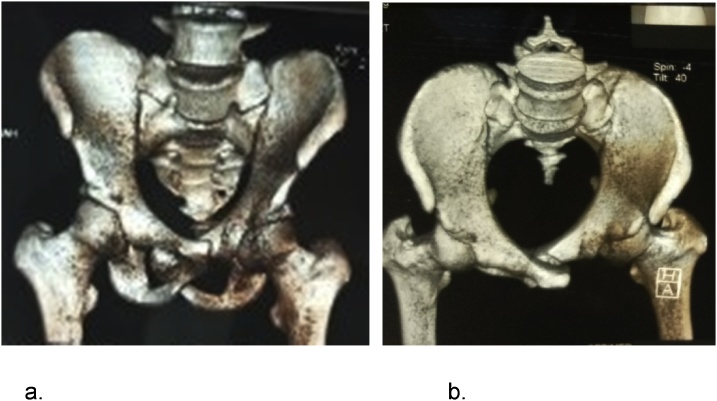
Pelvic CT scan showing overlapped pubic symphysis, with the left pubic symphysis locked into the right obturator foramen. a. Front view. b. Top view.

**Fig. 2:**
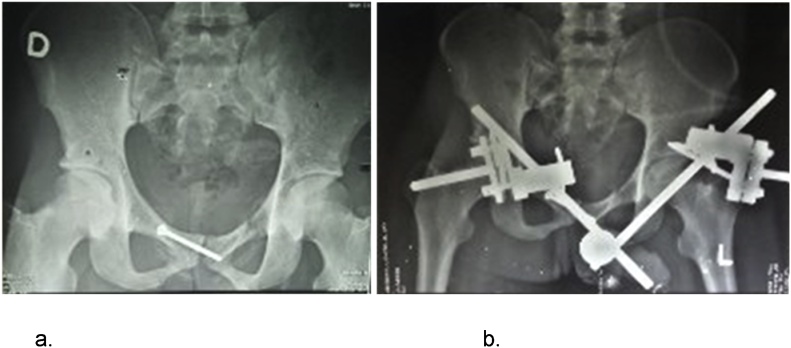
a. Trans-symphyseal screw. b. Stabilisation of the pelvic with Hoffman 2 external fixator.

We proceed with fluid resuscitation with normal saline, analgesics and suprapubic catheterization. The contrast pelvic CT Scan (Fig. 1) showed a pubic symphysis disjuncture with both pubic bones overlapping, the left posteriorly displaced pubic symphysis in the right obturator foramen, entrapped by the left pubic tubercle consistent with a locked symphysis fracture; which we classified as Tile B2 fracture. A surgical procedure was carried out on spinal anesthesia, one week after admission. The patient was lying supine, on an orthopedic table. An infra-umbilical median incision was done. Patient underwent an open pelvic reduction with internal and external fixation. The intraoperative findings were pubic symphysis disjuncture with a posteromedial displacement of the left pubic tubercle into the right obturator foramen where it was entrapped. The manual reduction was impossible; we then proceed with osteotomy of left superior pubic ramus to reduce the disjunction, which was successful. The reduction was stabilised using a trans-symphyseal screw of 60 mm × 3.5 mm, then external fixation with Hoffman 2 external fixator (Fig. 2).

**Fig. 3:**
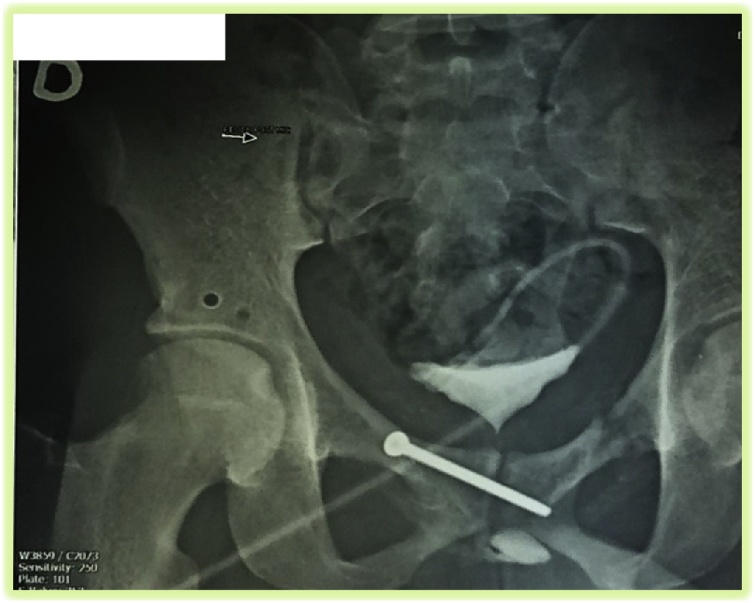
Combined antegrade and retrograde cystourethrography showing a non-patent stenosis of the initial segment of the posterior urethra.

Post-operative therapy comprises analgesic using tramadol and paracetamol. Thrombo-embolic prevention was done using 4000IU daily subcutaneous enoxaparin. Post-operative period was uneventful. On his 45th postoperative day a pelvic control x-ray showed an aligned public symphysis with trans-symphyseal screw in place. This enable us to remove the external fixator. From there he did also a retrograde and antegrade cystourethrography which showed a non-patent stenosis of the initial segment of the posterior urethra (Fig. 3). The patient proceeds with rehabilitation and we then referred him to the urologic clinic for management of his urethral injury. The delay between the admission of the patient and the surgical procedure was due to financial difficulties in the management, because of lack of adequate health insurance coverage rate.

## 3 Discussion

Locked pubic symphysis (“locked book pubic symphysis”) also referred to as overlapping pubic symphysis dislocation are very rare forms of pelvic injuries, with incidence and prevalence unknown. These injuries are more common in men than in women with the male to female ratio estimated at 11:3 [9]. According to Cannada and Reinert [5], the female pubic symphysis has a thicker cartilaginous disc and 2–3 mm greater mobility than the male pelvis contributing to it stability compared to the male pelvis. The mechanism of injury is not fully understood. However, most authors agree that this injury results from a lateral compressive force to the pelvis. From then on, it is postulated that this is combined with forced hyperextension and adduction of the hip [9]. This was the case in our patient as he reports being compressed on his left side by a fast-moving truck against a stationary truck. Eggers, in 1952 stated that the symphysis locked can either be anterior or posterior [3]. Most reported cases in literature are locked posteriorly [2,4,[10], [11], [12]], as in our case. Most reported cases of locked pubic symphysis have been hemodynamically stable. This is in keeping with the mechanism of lateral compression [9].

Urogenital injuries are the most common associated injuries to pelvic fractures in general [13]. These injuries have been reported to be associated with most locked pubic symphysis described in literature [2,4,[10], [11], [12]]. The case described herein had an initial urethral injury which was suspected by acute urinary retention. Attempt of transurethral catherization done in the dispensary where the patient initially presented probably worsen his urethral injury. We remain limited in the discussion of the management of the stenosis of the initial segment of the posterior urethra and its outcome, because of lack of enough data and hindsight.

Currently, there exist no standard classification for overlapping pelvic injuries as these injuries were not included by Young and Burgess [14]. They did not include this type of injury in their landmark article on classification of pelvic ring injuries. An attempt to correlate this, using Tile classification, classified the index patient as Tile B2.

The primary goal of treatment of overlapping public symphysis is to achieve and maintain reduction of the pubic symphysis. This can be done either by closed or open reduction. Few cases of successful closed reduction are described in literature [[15], [16], [17]]. Indication for open reduction include failure of closed reduction and severe disjunction of the public symphysis with a gross overlapped. Recently, open reduction has been described as the treatment of choice of overlapping pubic symphysis [4,12,18]. In our case reduction was impossible. We therefore performed an osteotomy of the left iliopubic ramus in order to ease reduction, with successful outcome. Iatrogenic osteotomy of iliopubic ramus to ease reduction of overlapping pubic symphysis has been described in the literature [19].

The reduction was stabilised using a trans-symphyseal screw of 60 mm × 4.5 mm, then external fixation with Hoffman 2 external fixator. Treatment of trans-symphyseal instability using an internal plate fixation is the most common procedure described in the literature [20]. Use of external fixator is described as a temporal treatment in major pelvic trauma or in case of polytrauma, when internal fixation is impossible. But in our case, we used external fixation as definitive treatment. We opted for an association of external fixation and internal fixation by using a trans-symphyseal screw for a better stability of the pelvis, since a single screw was used instead of a screwed plate. The choice of internal fixation by a screw instead of a screwed plate was motivated by higher risk of infection, considering a prior suprapubic catheterization done few days before. Suprapubic catheterization and delayed close stabilisation of the pelvis in such cases, is common in low-incomes settings. This may contraindicate the use of a screwed plate in order to stabilise the pelvis, because of higher infectious risk. In the previous description, close reduction has been attempted. But when unsuccessful, open reduction should be undertaken.

Usually, the urethral lesions are observed in anteroposterior compression of the pelvis by a shear mechanism. But, in this case, the compression was lateral, with stenosis of the initial segment of the posterior urethra. Associated urethral lesions may influence the choice of stabilisation. The delay between the admission of the patient and the surgical procedure was due to financial difficulties in the management, because of lack of adequate health insurance coverage rate.

## 4 Conclusion

Locked pelvic symphysis is a rare form of pelvic injury. The majority of cases require stabilization by open reduction and internal fixation of the anterior pelvic ring. Urogenital injuries are the commonest associated injuries, despite unfavourable mechanism.

## Funding

No funding sources.

## Ethical approval

Our study is exempt from ethical approval by the ethics committee of the Faculty of Medicine and Biomedical Sciences of the University of Yaoundé I (Cameroon).

## Consent

Written informed consent was obtained from the patient for publication of this case report and accompanying images. A copy of the written consent is available for review by the Editor-in-Chief of this journal on request.

## Author’s contribution

The patient was admitted and operated under the care of Marc Leroy GUIFO who concepted the study with Olivier Kopong FOLA. Jean Gustave TSIAGADIGUI and Paul TOLEFAC collected data. From there, the paper was written. Marcella BIYOUMA and Idris DJOKO reviewed the paper. Arthur ESSOMBA gave the final approval.

## Registration of research studies

N/A.

## Guarantor

**Arthur ESSOMBA**, Professor of General Surgery at the Faculty of Medicine and Biomedical Sciences of the University of Yaoundé I (Cameroon).

## Provenance and peer review

Not commissioned, externally peer-reviewed.

## Declaration of Competing Interest

The authors declare no conflict of interest.
